# Childbirth preparation is associated with self-efficacy and the use of non-pharmacological pain relief during labour

**DOI:** 10.1038/s41598-026-63451-w

**Published:** 2026-08-14

**Authors:** Patrycja Malinowska, Matylda Sierakowska

**Affiliations:** Department of Integrated Medical Care, Medical University of Bialystok ul. M. Skłodowskiej – Curie, Białystok, 7 A 15-096 Poland

**Keywords:** Labour, Childbirth preparation, Birth classes, Birth satisfaction, Health care, Medical research

## Abstract

Despite growing access to prenatal education, many women still report high levels of stress and dissatisfaction with their childbirth experience. Preparing for natural includes prenatal education, physical preparation of the body, and emotional support, which together help reduce anxiety, stress, and pain associated with childbirth. This study aimed to assess the extent of childbirth preparation among pregnant women and to evaluate how preparation impacts perceived stress, use of non-pharmacological pain relief methods, self-efficacy, and childbirth satisfaction. Cross-sectional studies were conducted from June 2023 to February 2024 with a group of 201 pregnant women at the University Clinical Hospital in Białystok in the Delivery Room and the postpartum ward. Participants completed questionnaires before and after birth. The study was conducted by means of a self-designed questionnaire, the Perceived Stress Scale (PSS-10), the Generalised Self-Efficacy Scale (GSES), and the Health Behaviour Inventory (HBI). No effect of childbirth preparation on perceived satisfaction after childbirth has been found. Women with a higher sense of self-efficacy more frequently used non-pharmacological methods of pain relief during childbirth, performed pelvic floor muscle exercises, used perineal massage, and prepared a birth plan. Childbirth preparation is not related to the mode of birth, but it increases the likelihood of using non-pharmacological methods of pain relief during childbirth. While childbirth preparation improves physical readiness and psychological resilience, satisfaction with childbirth is more strongly influenced by self-efficacy and previous childbirth experiences than by preparation alone. It is important for pregnant women to have access to appropriate sources of support and information, which may have a positive impact upon their birthing experience.

## Introduction

Each pregnant woman has different expectations regarding childbirth and its course. Preparing pregnant women for natural childbirth is an important factor that determines effective cooperation with medical staff in the delivery room as well as satisfaction with the childbirth experience^1^. Preparation not only improves physical condition but also reduces perceived pain and anxiety during childbirth^2^. Additionally, a pregnant woman who prepares herself for childbirth is characterized by a greater self-confidence and a stronger sense of decision-making and empowerment^3^.

The concept of childbirth preparation has evolved over many decades, beginning with the work of Grantly Dick-Read and later adapted in different countries, including Poland through the work of Włodzimierz Fijałkowski. These concepts emphasize reducing fear, improving knowledge, and promoting active participation during childbirth^4,5^.

Preparing for childbirth by a pregnant woman may take place on multiple levels, including participation in childbirth classes, where practical and theoretical sessions are conducted by midwives and obstetricians. In addition to gaining knowledge about pregnancy physiology, childbirth, and newborn care, pregnant women engage in physical exercises to enhance their body’s fitness and endurance. As part of childbirth classes, expectant mothers may visit delivery rooms where they intend to give birth. Furthermore, those classes are not exclusive to pregnant women; partners or husbands planning to support the woman during childbirth and postnatal care may also attend^5^. Pregnant women, in addition to attending childbirth classes, may benefit from prenatal visits provided by community midwives. During those visits, pregnant women have the opportunity to ask any questions they may have and to benefit from the knowledge of experienced community midwives, which may help reduce anxiety during childbirth^6^.

Among other skills that contribute to the preparation of a pregnant woman for childbirth, we may include perineal massage and Kegel exercises. Perineal massage stimulates nerve endings in the skin, improves blood circulation in the perineal tissues, enhances their elasticity and flexibility, and reduces the risk of perineal injuries during natural childbirth^7^. Kegel exercises are designed to strengthen the pelvic floor muscles, improve circulation, and reduce swelling in the perineal area^8^.

In addition to the aforementioned aspects essential to the preparation of a pregnant woman for childbirth, the possibility of creating an individual Birth Plan is worth mentioning. Engaging in the creation of such a document plays a crucial role in enhancing the pregnant woman’s sense of empowerment and decision-making. A Birth Plan allows for the expression of expectations and preferences. The World Health Organisation (WHO) recommends birth planning as part of prenatal care, which may minimise medicalisation during childbirth, facilitate communication between the pregnant woman and medical staff, and strengthen the woman’s position as the decision-maker regarding her own birth process^9^.

Therefore, this study aims to explore the knowledge and skills of women in preparation for natural childbirth and to investigate the relationships between the level of childbirth preparation and satisfaction with the birth experience. In particular, limited attention has been given to the relationships between childbirth preparation, psychological factors such as stress and self-efficacy, and satisfaction with the birth experience.

Despite the extensive body of research on prenatal education and childbirth preparation, most studies have focused primarily on clinical outcomes such as mode of delivery, pain perception, or childbirth satisfaction. Much less attention has been paid to the underlying psychological mechanisms that may explain these outcomes, particularly the role of self-efficacy.

The aim of this study was to examine the relationship between childbirth preparation and self-efficacy as a key psychological factor, and to assess how this relationship, together with other psychological and behavioural variables (such as perceived stress and health behaviours), influences childbirth-related behaviours and outcomes among pregnant women. In particular, the study addresses the insufficiently explored link between the need for childbirth preparation and women’s perceived self-efficacy.The following research hypotheses are posited:

- Attendance at childbirth classes will be associated with lower levels of perceived stress (PSS-10).
- Engagement in various forms of childbirth preparation will be associated with childbirth satisfaction.
- Higher scores on the Generalised Self-Efficacy Scale (GSES) will be positively associated with higher self-assessed childbirth preparation and greater engagement in childbirth preparation behaviours (e.g., birth plan preparation, pelvic floor exercises, and perineal massage).
- Higher scores on the Health Behaviour Inventory (HBI) will be positively associated with the level of childbirth preparation (self-assessed).
- Attendance at childbirth classes will be associated with greater knowledge of non-pharmacological pain relief methods and vertical birth positions.

## Materials and methods

### Participants

The research was conducted in a group of pregnant women at the University Clinical Hospital in Białystok, at the Perinatology and Obstetrics Clinic with a Childbirth School. The clinic is a tertiary care center providing specialized obstetric services. The study was carried out in two stages: before childbirth - in the delivery room, and postpartum - in the obstetrics department.

A formal sample size calculation was not performed prior to the study. The sample size was determined by the number of eligible participants available during the study period.

A convenience sampling method was used. This approach was appropriate due to the clinical setting and the accessibility of participants during the study period. Initially, 201 pregnant women admitted to the Delivery Room with full-term pregnancies (between completed 37th and 42nd weeks of gestation) were invited to participate in the study. Of these, 201 women completed Stage I of the study.

In Stage II, 122 participants completed the postpartum questionnaire. The final sample included 122 women who completed both stages of the study and were included in the analysis.

A reduction in sample size between Stage I and Stage II was observed. The main reasons for attrition included incomplete questionnaires, withdrawal from the study, lack of follow-up after delivery, and delivery in another healthcare facility. Due to limited availability of data on non-respondents, a full attrition analysis could not be performed; however, the potential impact of sample loss has been considered in the interpretation of the results.

The inclusion criteria were:

1. Pregnant women at term (37–42 weeks of gestation).
2. Admission to the delivery room.
3. Ability to provide informed consent.
4. Completion of the Stage I questionnaire.

The exclusion criteria were:

1. Incomplete questionnaires.
2. Withdraw from participation.
3. Lack of follow-up in stage II.

### Study design and data collection

The cross-sectional study was conducted from June 2023 to February 2024 by means of a diagnostic survey method. Patients received a questionnaire consisting of two parts: the first part to be completed in the Delivery Room and the second part to be completed in the postpartum ward. Participation in the study was voluntary and anonymous. Participants could withdraw from the study at any time. Participation in the study implied the consent to take part in the research.

### Measures

For the study purposes, the following Research tools have been applied:

#### Stage I

- A self-constructed questionnaire (31 questions) containing a record sheet (6 questions) as well as questions inter alia about: past deliveries, attendance at childbirth classes, preparation of a birth plan, assessment of the level of preparation for childbirth by the pregnant woman (1 – lack of knowledge and skills, 2 – rather unprepared, 3 – average preparedness, 4 – well-prepared, 5 – very well-prepared) and a partner/husband, knowledge of non-pharmacological methods of pain relief during childbirth, knowledge of vertical positions.
- Perceived Stress Scale PSS-10 by Cohen, Kamerck, Mamelstein^10^.

The scale consists of 10 items rated on a 5-point Likert scale (0–4). Positively worded items (items 4, 5, 7, and 8) were reverse coded prior to analysis. The internal consistency of the PSS-10 in the present study was α = 0.84, indicating good reliability and being consistent with values reported in previous validation studies (0.78–0.91).

#### Stage II

- A self-constructed questionnaire containing 25 questions about: a method of delivery, episiotomy, level of satisfaction with the childbirth (1 – none, 2 – poor, 3 – average, 4 – fairly high, 5 – high), application of non-pharmacological methods of pain relief during childbirth and vertical positions, influence of preparation for childbirth on relations with medical personnel and perception of childbirth.
- Generalised Self-Efficacy Scale (GSES) by Schwarzer, Jerusalem, Juczyński^11^.

The scale consists of 10 items, with higher scores indicating greater perceived self-efficacy. In the present study, the internal consistency of the GSES was α = 0.86 which is comparable to values reported in previous studies (0.76–0.90).

- Health Behaviour Inventory (HBI) by Juczyński^12^.

The scale consists of 24 items. In the present study, the internal consistency of the HBI was α = 0.81, indicating good reliability. This result is consistent with the values reported in previous validation studies.

The self-designed questionnaires used in Stages I and II were developed based on a review of the literature concerning prenatal education, childbirth preparation, and maternal childbirth experiences, as well as current recommendations regarding perinatal care. The content of the questionnaires was reviewed by academic staff with expertise in midwifery and obstetrics to ensure content validity. The questionnaires consisted primarily of factual and descriptive items; therefore, internal consistency measures such as Cronbach’s alpha were not applicable.

All values exceeded the acceptable threshold of 0.70, indicating satisfactory internal consistency of the scales.

*The Perceived Stress Scale PSS-10* by Cohen, Kamerck, Mamelstein is a research tool consisting of 10 questions regarding various subjective feelings related to personal events, problems, and coping methods. Participants respond to statements by entering a number from 0-never to 4-very often. The overall score is the sum of the points counted, with a theoretical distribution ranging from 0 to 40. A higher score indicates a greater intensity of perceived stress. After transformation into standardised units, the overall index is interpreted according to the properties characterising the sten scale. As far as the sten scale is concerned, scores within 1–4 sten are considered *low*, scores within 5–6 sten are considered *average*, and scores within 7–10 sten are considered *high*^10^.

*The General Self-Efficacy Scale (GSES)* by R. Schwarzer, M. Jerusalem, Z. Juczyński, is a research tool consisting of 10 questions designed to measure an individual’s general belief in the ability to cope effectively with various difficulties and obstacles. Participants respond to statements on a 4-point scale. The sum of all points provides a total score ranging from 10 to 40. The overall score, after transformation into standardised units, is subject to interpretation. Scores within 1–4 sten are considered *low*, scores within 5–6 sten are considered *average*, and scores within 7–10 sten are considered *high*^11^.

*The Health Behaviour Inventory HBI* by Juczyński consists of 24 statements describing positive health-related behaviour. Those statements are divided into four categories: proper dietary habits, preventive behaviour, positive psychological attitudes, and health practices. Participants respond to the statements on a 5-point scale, ranging from 1-almost never to 5-almost always. As far as the sten scale is concerned, scores within 1–4 sten are considered *low*, scores within 5–6 sten are considered *average*, and scores within 7–10 sten are considered *high*^12^.

### Ethics consideration

The research was carried out in accordance with the Declaration of Helsinki and Good Clinical Practice in research. The Bioethics Committee of the Medical University in Bialystok, Poland granted the ethical approval for the study (APK.002.180.2023, APK.002.24.2024.). Participation was voluntary and participants were informed about the project.

### Statistical analysis

- The obtained results were subjected to the statistical analysis in which the arithmetic mean and standard deviation were calculated, and the minimum and maximum values for quantitative variables were presented. The percentage distribution was calculated for qualitative variables. The relationships between pairs of quantitative variables were analysed using the Spearman’s rank correlation coefficient. For nominal characteristics, the significance of differences between groups was assessed using the Mann-Whitney U test or the chi-square independence test.
- A significance level of *p* < 0.05 was considered statistically significant; *p* < 0.01 was considered highly significant; and *p* < 0.001 was considered very highly statistically significant. The data were processed in Microsoft Excel 2013 and analysed using the Statistica v.13 software package.

## Results

### General characteristics of the study group

The analysis of women’s preparation for childbirth was conducted among 201 women (Stage I of the study), while only 122 respondents participated in the studies concerning the course of childbirth (Stage II). Comparative analyses of the pre-birth period (Stage I) with the post-birth period (Stage II) were conducted in respect of a group of 122 patients who had completed the questionnaires both before and after childbirth.

Most participants were aged between 22 and 40 years, had higher education, lived in urban areas, and were married or in a relationship (Table 1).

**Table 1:** General characteristics of the study group.

Variable	*N* (%)
Age [Year]	
under 22	2 (1.0%)
22–30	109 (54.2%)
31–40	87 (43.3%)
Over 40	3 (1.5%)
Socioeconomic status	
Very Good	50 (24.9%)
Good	89 (44.3%)
Fairly Good	62 (30.9%)
Educational background	
Advanced Education	122 (60.7%)
Bachelor’s Degree/Engineering	30 (14.9%)
Secondary Education	49 (24.4%)
Marital status	
Single	15 (7.5%)
Married/In Relationship	183 (91.0%)
Divorced	3 (1.5%)
Place of residence	
Rural Area	54 (26.9%)
City with a Population of 50,000 to 100,000	37 (18.4%)
City with a Population of 100 thousand	110 (54.7%)
Professional activity	
Professionally Active	93 (46.3%)
Unemployed	21 (10.4%)
On a Sick Leave/On a Holiday Leave	87 (43.3%)
Children	
None	134 (66.7%)
Yes, One	44 (21.9%)
Yes, Two	17 (8.5%)
Yes, More Than Two	6 (3.0%)
Previous natural childbirth	
Yes	59 (29.4%)
No	142 (70.6%)

## I stage of study – pre-birth period analysis

### Preparation for childbirth

Approximately half of the women attended childbirth classes, whereas participation in antenatal education provided by community midwives and visits to the delivery room were less common. Most participants prepared a birth plan and rated their level of childbirth preparation as moderate or high (Table 2).

**Table 2:** Preparation of pregnant women for childbirth.

Variable	Number (*n* = 201)	Percentage Share
Attendance at childbirth classes during the pregnancy		
Yes	97	48.3%
No	95	47.3%
Sporadically	9	4.5%
Visit to a urogynecological physiotherapist during the pregnancy		
Yes	48	23.9%
No	142	70.6%
Sporadically	11	5.5%
Reading books about childbirth written by midwives or doctors		
Yes	133	66.2%
No	68	33.8%
Visiting the delivery room before childbirth		
Yes	19	9.5%
No	182	90.5%
Taking online courses preparing for childbirth		
Yes	66	32.8%
No	132	65.7%
Lack of knowledge	3	1.5%
Attending antenatal education conducted by a community midwife		
Yes	79	39.3%
No	109	54.2%
Sporadically	13	6.5%
Regular pelvic floor muscle exercises during pregnancy		
Yes	75	37.3%
No	126	62.7%
Perineal massage during pregnancy		
Yes	37	18.4%
No	122	60.7%
Sporadically	42	20.9%
Using supplementation with evening primrose oil or raspberry leaf tea		
Yes	52	25.9%
No	138	68.7%
Lack of knowledge	11	5.5%
Creating a birth plan		
Yes	132	65.7%
No	63	31.3%
Lack of knowledge	6	3.0%
Assessment of preparedness for childbirth		
Lack of knowledge and Skills	3	1.5%
Rather Unprepared	16	8.0%
Average Preparedness	78	38.8%
Well Prepared	91	45.3%
Very Well Prepared	13	6.5%

The analyses demonstrated that age did not differentiate the use of various forms of childbirth preparation. All comparisons yielded test probabilities (p-values) significantly exceeding 0.05.

Differences according to educational background were observed. The following results have been achieved:

- Women with lower educational background significantly less frequently participated in childbirth education classes (*p* = 0.0041**);
- Women with secondary educational background significantly less frequently used books about childbirth (*p* = 0.0004***);
- Lower education was associated with less frequent use of pelvic floor muscle exercises (*p* = 0.0094**), perineal massage (*p* = 0.0434*) and online course attendance (*p* = 0.0438*);

Women from large cities, as compared to those from rural areas and small towns, significantly more often attended childbirth classes (*p* = 0.0069**), were more frequently educated by community midwives (*p* = 0.0149*), and used perineal massage during pregnancy (*p* = 0.0137*).

Previous natural childbirth was associated with lower participation in childbirth classes (*p* = 0.0000***), reading books (*p*=0.0001***), preparing a birth plan (*p* = 0.0015**), and using perineal massage (*p* = 0.0061**).

Attending childbirth classes is almost unrelated to the self-assessment of preparation for childbirth (*p* = 0.2102).

Women attending childbirth classes rated their preparation significantly higher for specific aspects of childbirth preparation, namely the use of vertical positions (*p* = 0.0000***) and non-pharmacological pain relief methods during labour (*p* = 0.0000***). Attendance at childbirth classes was not associated with overall self-assessment of childbirth preparation (*p* = 0.210). However, it was significantly associated with higher preparedness for the use of vertical positions and non-pharmacological pain relief methods (*p* < 0.001) (Table 3).

**Table 3:** Childbirth preparation in relation to attending childbirth classes.

Outcome	No classes (%)	Classes (%)	*p*-value
Well/very well prepared-vertical positions	19.3	44.3	< 0.001
Well/very well prepared-non-pharmacological pain relief	25.9	52.6	< 0.001

### Perceived antenatal stress

In order to assess the level of stress, a standardised questionnaire PSS-10 was used. The results may range from 0 to 40 points, where a higher score indicates a higher level of stress. Most participants reported low or moderate stress levels, while approximately one-quarter experienced high perceived stress (Table 4).

The detailed analysis of the research results has revealed that women with high levels of stress less frequently attended childbirth classes (28.0%) as compared to women with low and moderate levels of stress (54.1% and 56.1%, respectively) (*p* = 0.0041**). Conversely, as far as visits to urogynecological physiotherapists are concerned, it has been found that women with low levels of stress paid those visits most frequently, while those with moderate stress levels did so the least (33% vs. 14%) (*p* = 0.0208*).

Stress level was associated with parity (*p* = 0.0003). Among the respondents with high levels of stress, only 17.2% had no children, 29.5% had one child, and as many as 60.9% had two or more children (*p* = 0,0003***) (data in Table 4).

**Table 4:** The level of perceived stress (PSS-10) and attending childbirth classes, visiting a urogynecological physiotherapist, and having children.

Stress level		Number						Percentage Share
Low stress		85						42.3%
Moderate stress		66						3.8%
High stress		50						24.9%

Stress level								
Childbirth Preparation Form		Low stress		Moderate stress		High stress		*p*-value
		*N*	%	*N*	%	*N*	%	
Attending Childbirth Classes		46	54.1%	37	56.1%	14	28.0%	0.0041**
Visit to a urogynecological physiotherapist during the pregnancy		28	32.9%	9	13.6%	11	22.0%	0.0208*

Stress level	Children (*p* = 0,0003***)						Total	
	none	yes, one		yes, two or more than two				
Low stress	65 (48.5%)	16 (36.4%)		4 (17.4%)			85	
Moderate stress	46 (34.3%)	15 (34.1%)		5 (21.7%)			66	
High stress	23 (17.2%)	13 (29.5%)		14 (60.9%)			50	
Total	134	44		23			201	

### II stage of study – post-birth period analysis

#### Characteristics of the study group

The analysis covers 122 women from the total study population (*n* = 201) who completed surveys before and after childbirth. The majority of respondents (70.5%) gave birth vaginally without any episiotomy (67.4%). Nearly three-quarters of the women responded that they were satisfied with the course of their childbirth (‘yes’ − 37.7%, ‘rather yes’ − 36.1%).

### Preparation for childbirth and vaginal delivery versus episiotomy

There was no significant relationship found between childbirth preparation and the type of delivery (vaginal birth or cesarean section) (test probability values *p* above 0.05).

A significant association with the occurrence of an episiotomy was observed in cases where pregnant women participated in education provided by a community midwife. Community midwife-led education was associated with episiotomy status (*p* = 0.0292). Avoidance of episiotomy was reported in 53.1% of women who received such education and 75.9% of those who did not. Pelvic floor muscle exercises during pregnancy were also associated with avoidance of episiotomy (80.6% vs. 58.0%, *p* = 0.0277*)(Table 5).

**Table 5:** Childbirth preparation versus episiotomy.

Childbirth Preparation Form	Number and percentage of cases avoiding episiotomy depending on childbirth preparation					*p*-value
	No		Yes			
	*n/N*	%	*n/N*	%	OR	
Attending antenatal education conducted by a community midwife	41/54	75.9%	17/32	53.1%	0.36 (0.14-0.91)	0.0292*
Regular pelvic floor muscle exercises during pregnancy	29/50	58.0%	29/36	80.6%	3.00 (1.11-8.14)	0.0277*

N - totality of women giving birth vaginally.

### Satisfaction with childbirth experience

The mean satisfaction with childbirth experience was calculated on a 5-point scale (1–5). No statistically significant associations were found between the use of various forms of childbirth preparation and satisfaction with childbirth (all *p* > 0.05) (Table 6).

Previous vaginal childbirth was associated with higher satisfaction scores (4.54 vs. 3.73; *p* < 0.001). Satisfaction also increased with the number of children, with mean scores of 3.73 for women without children, 4.28 for those with one child, and 4.65 for those with two or more children (*p* < 0.001) (Table 6).

**Table 6:** The satisfaction level with childbirth experience in relation to past births and having children.

Variable	Mean satisfaction (SD)	*p*-value
Previous vaginal childbirth		
Yes	4.54	
No	3.73	< 0.001
Number of children		
None	3.73	
One	4.28	
Two or more	4.65	< 0.001

Mean values are presented. Statistical significance was assessed using the Mann-Whitney test and Kruskal-Wallis test.

### Preparing your partner for childbirth

Most women reported that their partner’s knowledge and skills were sufficient (81.1%). The study examined how the assessment of the partner’s attitude after childbirth depended on the evaluation of their participation in childbirth preparation during pregnancy. Partner participation in childbirth preparation was associated with postnatal assessment of partner knowledge and skills (*p* = 0.0004***).

### The use of non- pharmacological methods for pain relief during childbirth

The use of non-pharmacological pain relief methods was significantly associated with prior knowledge of these methods. Women who were familiar with specific techniques were more likely to use them during childbirth (all *p* < 0.05), except for warm compresses (*p* = 0.107) (Table 7).

**Table 7:** Methods used for pain relief and knowledge of those methods.

Method	OR (95%CI)	*p*-value
Massage	5.47 (1.54–19.41)	0.0042**
Aromatherapy	10.83 (1.31–89.54)	0.0072**
Music therapy	3.98 (1.46–10.83)	0.0047**
Vertical positions	5.42 (2.27–12.95)	0.0001***
Correct breathing techniques	3.46 (1.27–9.43)	0.0119*
Water immersion	5.30 (1.41–19.89)	0.0072**
Warm compresses	2.06 (0.85–4.99)	0.1072
TENS	3.65 (1.00–13.26)	0.0388*

### Feelings regarding the course of childbirth

Feelings regarding the course of childbirth are quite varied - approximately 60% indicate positive responses, while the remaining 40% are negative. Among those, unequivocally positive and negative responses each constitute about one-third of the total responses in their respective groups.

The relationship between previous childbirth experiences and the evaluation of childbirth in terms of expectations has been examined. Due to the ordinal nature of both characteristics, the Spearman’s rank correlation coefficient was used for assessing the relationship.

The number of children was correlated with evaluation of childbirth (rS = 0.39; *p* = 0.0000), and previous vaginal childbirth was associated with evaluation of childbirth in terms of expectations (*p =* 0.0000***).

### Childbirth preparation and evaluation of generalised self-efficacy (GSES)

More than a half of the surveyed women (53.3%) have been classified, according to the GSES scale, as having a high sense of self-efficacy. The average self-efficacy score in the studied population has been 30.0 points (range 10–40 points). A half of the obtained results have been grouped in a narrow range of 28–32 points, based on the values of the 25th and 75th percentiles. A low self-efficacy (10–24 points, 1–4 stens) characterises 10 respondents, 47 shows an average self-efficacy (25–29 points, 5–6 stens), and the majority, 65 women, indicate a high self-efficacy (30–40 points, 7–10 stens).

Higher self-efficacy was associated with greater engagement in childbirth preparation behaviours and higher self-assessed childbirth preparation (Table 8).

**Table 8:** Childbirth preparation versus Generalised Self-efficacy (GSES).

GSES					
Childbirth Preparation Form	No		Yes		*p-value*
	*N*	$\overline{x}$	*N*	$\overline{x}$	
Pelvic floor exercises during pregnancy	71	29.0	51	30.5	0.0205*
Perineal massage during this pregnancy	92	29.3	30	30.6	0.0284*
Preparing a birth plan	41	28.6	81	30.1	0.0119*

Self-assessment of childbirth preparation													
	Low (*N* = 12)			Average (*N* = 44)			Well (*N* = 56)			Very Well (*N* = 10)			*p*-value
GSES	$\overline{x}$	Me	*s*	$\overline{x}$	Me	*s*	$\overline{x}$	Me	*s*	$\overline{x}$	Me	*s*	0.0236*
	28.9	29	3.5	28.6	29	3.8	30.0	30	3.9	32.6	33.5	3.9	

### Childbirth preparation and health behaviour of participants (HBI)

Respondents answering 24 statements in the HBI scale scored between 55 and 104 points. The arithmetic mean score in the HBI was 84.0. Among those surveyed, one-fourth had a low level of health behaviour (1–4 sten), one-fourth had a high level (7–10 sten), and almost exactly half had an average level (5–6 sten). The highest ratings were for positive psychological attitudes, while the lowest were for correct dietary habits (averaging 3.71 and 3.32, respectively).

The Mann-Whitney test was used for assessing the impact of various forms of childbirth preparation on the level of health behaviour.

During the analysis, the percentage share of women engaging in various forms of childbirth preparation was compared based on the classification of health behaviour. The significance of differences between groups of women with different levels of health behaviour in adopting specific forms of childbirth preparation was assessed using the chi-square independence test.

Women with higher HBI scores were more likely to engage in several childbirth preparation activities, particularly attendance at childbirth classes, reading educational materials, and preparing a birth plan (Table 9).

**Table 9:** Childbirth preparation and HBI – Health Behaviour Inventory.

Childbirth Preparation Form	Mean HBI	*p*-value
Attendance at childbirth classes during the pregnancy	86.5 vs. 81.1	0.009
Reading books about childbirth	86.5 vs. 79.2	< 0.001
Birth Plan Creation	85.7 vs. 80.4	0.002

## Discussion

The present study provides insight into how different forms of childbirth preparation relate to psychological and behavioural outcomes among pregnant women. Preparing for childbirth may occur on several levels. It’s important to have knowledge of basic theoretical and practical aspects as well as psychological preparedness. Practical knowledge and skills may be acquired through various preparatory methods. Most importantly, attending childbirth classes, reading guides and books written by professionals in this field, and receiving education from a midwife or attending physician are recommended. Such preparation aims to ensure the physical and psychological well-being not only of the pregnant woman but also her family^13^. It would seem that in Poland, there is easy access to opportunities for childbirth preparation. There are at least 400 registered childbirth schools, and educational visits conducted by midwives are reimbursed for all pregnant women^14,15^.

In our study, fewer than half of the participants attended childbirth classes. Similar associations between educational level and participation in prenatal education have been reported previously. Women with lower educational attainment and those living in rural areas were less likely to attend childbirth classes, suggesting that accessibility and health literacy remain important barriers to participation. Therefore, educating patients about the benefits of attending childbirth classes is crucial. Every pregnant woman should be aware that participating in childbirth classes not only reduces the rate of cesarean sections, preterm births, and scheduled inductions but also builds a sense of empowerment and self-confidence^16–18^.

The related literature reports indicate that vertical positions and other non-pharmacological pain relief methods serve the purpose of not only alleviating pain but also shortening the duration of the first and second stages of labour. They reduce the likelihood of obstetric complications, positively influence the psychoemotional state of the patient, and promote progress during childbirth^19–21^. In addition to benefits for the mother, it’s worth mentioning that vertical positions also have a positive impact on the foetus by relieving pressure on blood vessels^22^. Consistent with the fifth hypothesis, attendance at childbirth classes was associated with better preparation for the use of non-pharmacological pain relief methods and vertical positions during labour. This suggests that childbirth classes may play an important role in translating theoretical knowledge into practical coping strategies during childbirth. It’s important, as emphasised by other researchers, for the birthing patient to be aware of methods that may help reduce labor pain, as knowledge of those methods may positively influence satisfaction with the childbirth experience^23^.

Engaging in physical exercises and performing Kegel exercises during pregnancy brings many benefits. The World Health Organisation (WHO) recommends that women with uncomplicated pregnancies engage in 150 min of moderate-intensity physical activity per week. Many national and international guidelines emphasise the importance of incorporating physical activity into the daily routine of pregnant women^24,25^. Kegel exercises primarily strengthen the pelvic floor muscles, which may contribute to shortening the first and second stages of labour. Additionally, those exercises may help prevent urinary incontinence during pregnancy and in the postpartum period^26,27^.

In our study, it has been shown that only 37.3% of women regularly performed pelvic floor muscle exercises. In comparison, in similar postpartum studies conducted in the United States, as many as 65.5% of patients performed those exercises during pregnancy^28^. Given the benefits of performing Kegel exercises and attending visits with a urogynecologist physiotherapist, who is specialised in pelvic floor muscles.

Our own research has also revealed that 32.8% of patients used online childbirth preparation courses. That option is particularly convenient for pregnant women who live far from childbirth schools or have children requiring care. Other researchers have noted that women are increasingly interested in using online content about pregnancy as a source of knowledge^29^.

In prenatal care, it is important to emphasise the substantive value of content provided by qualified obstetrics professionals. Online childbirth preparation courses may be a particularly beneficial initiative for pregnant women living in small towns and rural areas where access to childbirth schools, midwives, or physiotherapists may be limited. The third hypothesis was supported, as higher self-efficacy scores were associated with higher self-assessed childbirth preparation and greater engagement in childbirth preparation behaviours. The fourth hypothesis was also supported, because higher health behaviour scores were associated with greater involvement in childbirth preparation.

Previous studies have reported inconsistent findings regarding the effect of prenatal education on childbirth satisfaction and mode of delivery. While some studies demonstrated higher maternal satisfaction and lower cesarean section rates, others—similarly to our findings—did not observe such associations. These discrepancies suggest that childbirth satisfaction depends on multiple clinical and psychological factors rather than prenatal preparation alone. Consistent with these observations, our findings did not support the second hypothesis, as no significant association was found between childbirth preparation and childbirth satisfaction^30–35^.

These findings underline the importance of individualized intrapartum care, as childbirth satisfaction appears to depend on multiple clinical and psychological factors. It would be important not only to ensure a proper level of preparation for patients before childbirth but also to provide adequate care during childbirth. It may influence the sense of empowerment and control over the childbirth process.

Our findings highlight the importance of self-efficacy in childbirth preparation. Women with higher self-efficacy were more likely to engage in childbirth preparation behaviours, including pelvic floor muscle exercises, perineal massage, birth plan preparation, and the use of vertical positions during labour. These findings indicate that higher self-efficacy was associated with greater engagement in childbirth preparation behaviours and the use of evidence-based practices during labour.

Similarly, women who perceived themselves as better prepared for childbirth reported higher levels of self-efficacy. Previous studies have suggested that childbirth self-efficacy is closely related to women’s psychological adjustment after childbirth and may influence the risk of birth-related psychological distress^36^.

The first hypothesis was supported - attendance at childbirth classes was associated with lower levels of perceived stress. In the studies presented, women who attended childbirth education classes and visited a urogynecological physiotherapist exhibited lower levels of stress. Therefore, it may be speculated that childbirth preparation carries many benefits, such as reducing perceived stress levels and more frequent use of non-pharmacological methods for pain relief during labour. These findings are consistent with recent systematic reviews demonstrating the beneficial effects of antenatal education on women’s psychological preparation for childbirth^37,38^. It should be noted, however, that childbirth preparation alone is not the key to a high satisfaction for women after childbirth.

In summary, given the goals of our research, we have shown that pregnant women generally feel adequately prepared for childbirth. As part of their preparation, they most commonly created a birth plan and used books authored by midwives and doctors, while they least frequently visited the delivery room before childbirth and used perineal massage. Women with a higher educational background, living in large cities, and without children were more likely to consciously prepare for childbirth. A significant observation from the study is that attending childbirth classes was associated with lower levels of stress whereas previous childbirth experiences were linked to higher levels of prenatal stress and satisfaction with childbirth. However, no relationship was found between childbirth preparation and satisfaction with childbirth. Furthermore, women with a higher sense of self-efficacy were more likely to use non-pharmacological pain relief methods during childbirth, perform pelvic floor exercises, use perineal massage, prepare a birth plan, and rate their level of preparation for childbirth higher. Most respondents exhibited an average level of health behaviour, and women with a higher health behaviour score more frequently prepared for childbirth. Preparation for childbirth was associated with a greater likelihood of using non-pharmacological pain relief methods and adopting vertical positions during labour, although no association with the mode of delivery was observed.

### Study constraints

These studies have their limitations which should be considered when interpreting the results. Firstly, they are cross-sectional studies based solely on self-assessment. Although standardised research tools used in this study are sensitive instruments designed to detect various states and traits, all responses focus on subjective feelings of the respondents rather than objective criteria, which poses a risk of false-positive results. Secondly, the study was conducted in two stages – before childbirth in the delivery room and after in the maternity ward. Only 60% of women completed the surveys in the second stage. Another limitation of this study is its single-centre design, which may limit the generalizability of the findings to other populations or healthcare settings. It could have influenced the results of comparative analyses, although the authors believe that despite these limitations, the studies are valuable for midwifery practice. The results may provide a basis for developing recommendations regarding education and care for pregnant and birthing women. Undoubtedly, the findings underscore the significant role of midwives in prenatal and perinatal care, influencing the satisfaction of pregnant women with their childbirth preparation and experience.

## Data Availability

The datasets used and analyzed during the current study available from the corresponding author on reasonable request.
